# Immunosuppression Induced by Glutamine Deprivation Occurs *via* Activating PD-L1 Transcription in Bladder Cancer

**DOI:** 10.3389/fmolb.2021.687305

**Published:** 2021-11-05

**Authors:** Liping Wang, Ting Xu, Xuecheng Yang, Zhijuan Liang, Jisheng Zhang, Dan Li, Yuanbin Chen, Guofeng Ma, Yonghua Wang, Ye Liang, Haitao Niu

**Affiliations:** ^1^ Key Laboratory, Department of Urology and Andrology, Medical Research Center, The Affiliated Hospital of Qingdao University, Qingdao, China; ^2^ Department of Geratology, The 971th Hospital of PLA Navy, Qingdao, China; ^3^ Department of Urology, The Affiliated Hospital of Qingdao University, Qingdao, China; ^4^ Department of Otolaryngology-Head and Neck Surgery, Key Laboratory, Medical Research Center, The Affiliated Hospital of Qingdao University, Qingdao, China

**Keywords:** glutamine deprivation, PD-L1, bladder cancer, T cells, MEK/ERK/c-Jun signaling pathway

## Abstract

Few studies have reported whether nutrients in the tumor microenvironment can regulate the expression of PD-L1. Since tumor cells are often situated in a low-glutamine environment, we investigated PD-L1 expression under glutamine deprivation in bladder cancer cells. PD-L1 expression and the activation of the EGFR/MEK/ERK/c-Jun signaling pathway under glutamine deprivation were investigated by qPCR, Western blot, and immunofluorescence analyses. C-Jun-mediated transcriptional regulation of the PD-L1 gene was assessed by ChIP. PD-L1 expression and activation of the EGFR/MEK/ERK/c-Jun signaling pathway were assessed in T24 cells, TCCSUP cells and BALB/c mice with or without glutamine supplementation. Additionally, the impact of PD-L1 expression under glutamine deprivation on the function of T cells was investigated by ELISA. The expression of PD-L1 and EGFR/MEK/ERK/c-Jun pathway activation were elevated by glutamine deprivation, and c-Jun was enriched in the enhancer region of PD-L1. The expression of PD-L1 was considerably impaired by inhibiting the EGFR/MEK/ERK/c-Jun pathway and was elevated by activating this signaling pathway. In addition, the elevated PD-L1 expression and MEK/ERK/c-Jun signaling pathway activation were reduced by glutamine supplementation *in vitro* and *in vivo*. PD-L1 upregulation by glutamine deprivation in bladder cancer cells could reduce IFN-γ production by T cells. The expression of PD-L1 was upregulated under glutamine deprivation through the EGFR/MEK/ERK/c-Jun pathway to impair T cell function.

## Introduction

Carcinoma of the urinary bladder has become a common cancer globally, and the treatments include surgery, chemotherapy and immunotherapy (Bellmunt et al., 2017; Liang et al., 2021). Suppression of checkpoint proteins has become the focus of modern immunotherapy (Bidnur et al., 2016). The most attentive checkpoint targets in bladder cancer treatments are programmed cell death protein-1 (PD-1), programmed death ligand-1 (PD-L1) and cytotoxic T lymphocyte-associated protein 4 (CTLA-4) (Sundararajan and Vogelzang 2015; van Hooren et al., 2017). Among them, PD-L1 has been shown to correlate with the severity and outcome of bladder cancer. It has been reported that PD-L1 overexpression is significantly associated with tumor grade and the postoperative prognosis of human urothelial cancers (Nakanishi et al., 2007; Boorjian et al., 2008), and PD-L1 can inhibit the function of T cells, such as inhibiting cytokine production, inducing cell apoptosis, and reducing T cell cytotoxicity (Dong et al., 2002; Iwai et al., 2002). However, most studies on PD-L1 have focused on clinical therapy in bladder cancer, and the regulatory mechanisms of PD-L1 overexpression need to be fully investigated.

It would be helpful to improve anti-PD-L1 treatments if the regulatory mechanism of PD-L1 expression in the tumor environment was fully understood. Studies have shown that various mechanisms can regulate the expression of PD-L1 (Boussiotis 2016). First, PD-L1 expression can be controlled by oncogenic pathways (Chen et al., 2016). The activation of EGFR can upregulate the expression of PD-L1 in non-small-cell lung cancer (NSCLC) (Akbay et al., 2013; Chen et al., 2015). PD-L1 expression can also be activated by the MAPK and PI3K-Akt pathways (Jiang et al., 2013; Chen et al., 2015). Many transcription factors have been reported to be involved in driving PD-L1 expression, such as HIF-1 and STAT3 (Marzec et al., 2008; Noman et al., 2014). The negative regulation of PD-L1 in cancer cells occurs by epigenetic mechanisms through microRNAs, including miR-197, miR-200, miR-513, miR-570 and miR-34a (Chen et al., 2016). In addition, the expression of PD-L1 is also regulated by proinflammatory cytokines in the tumor microenvironment, such as tumor necrosis factor-α (TNF-α), type I and type II interferons, and VEGF, among which interferon-γ is the most potent (Boussiotis 2016). However, few studies have reported whether changes in nutrient metabolism can drive PD-L1 expression in the tumor microenvironment.

Glutamine is one of the essential nutrients for cancer cell survival and proliferation. The dependence of tumor cells on glutamine metabolism makes it a potential anticancer target. Recent studies demonstrated that inhibitors of glutamine metabolism, expected to be an adjuvant to immunotherapy, can inhibit tumor progression through metabolism suppression and specific immunopotentiation, such as enhancing the immune function of CD8 + T cells in colon cancer and pancreatic ductal adenocarcinoma (Leone et al., 2019; Sharma et al., 2020). Glutamine also plays an important role in the development stage of bladder cancer. It has been reported that increased SLC1A5 (a glutamine transporter) expression and glutamine addiction occurr in long-term arsenite-induced human uroepithelial cells to study their malignant transformation (Li et al., 2021). Because of the poor vascularization at tumor sites and excessive consumption by tumor cells, glutamine is often deficient in the tumor microenvironment (Tran et al., 2017). The levels of glutamine have been reported to be lower in numerous tumors, including hepatomas and squamous cell carcinoma, than in normal tissues (Roberts and Frankel 1949). Thus, to grow and survive in conditions lacking glutamine, tumors have to develop various strategies (Reid et al., 2013; Tran et al., 2017; Ishak Gabra et al., 2018). To investigate whether glutamine deprivation affects PD-L1 expression in bladder cancer cells, glutamine was deprived from the culture medium of two highly malignant bladder cancer cell lines (T24 and TCCSUP cells) to detect PD-L1 expression and the signaling pathway involved in this event. Furthermore, we supplemented glutamine into *in vivo* xenografts of BALB/c mice and then measured changes in PD-L1 expression and activation of the associated signaling pathway. The effect on the function of T cells was also investigated.

## Results

### PD-L1 upregulation Was Induced by Glutamine Deprivation

**FIGURE 1:**
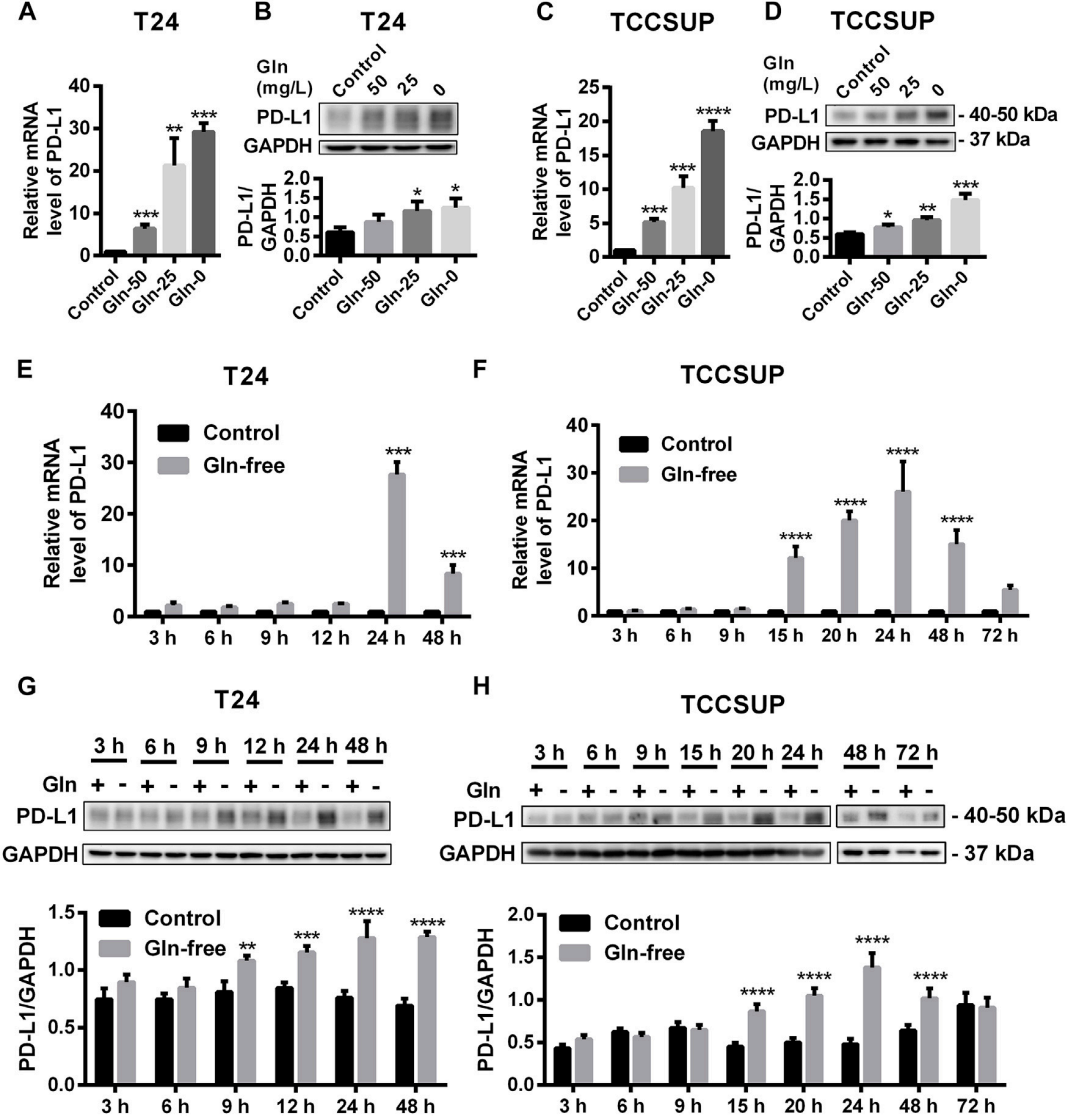
The expression of PD-L1 is upregulated by glutamine deprivation in T24 and TCCSUP cells. **(A,B)** The mRNA and protein levels of PD-L1 in T24 cells cultured in low-glutamine and glutamine-free medium (Gln 50, 25, 0: 50, 25, 0 mg/L glutamine; Control: 300 mg/L glutamine). **(C,D)** The levels of mRNA and protein for PD-L1 in TCCSUP cells cultured in low-glutamine and glutamine-free medium. **(E,G)** The mRNA and protein levels of PD-L1 in T24 cells after culturing in glutamine-free culture medium for 3–48 h **(F,H)** The mRNA and protein levels of PD-L1 in TCCSUP cells after culturing in glutamine-free culture medium for 3–72 h. The densitometric analysis of proteins was performed and the results were normalized to GAPDH. The results were considered significant at *p* < 0.05 (*p < 0.05, **p < 0.01, ***p < 0.001, ****p < 0.0001).

After T24 and TCCSUP cells were cultured in low-glutamine or glutamine-free culture medium (50, 25, 0 mg/L; control: 300 mg/L), the mRNA and protein levels of PD-L1 were determined by qPCR and Western blotting, respectively. The mRNA levels of PD-L1 in low-glutamine and glutamine-free medium were significantly higher than those in normal medium (Figures 1A,C), and the change in protein levels was consistent with that in the mRNA levels (Figures 1B,D). Furthermore, we investigated the mRNA levels of PD-L1 after glutamine deprivation at different time points. The qPCR results showed that the highest upregulation of PD-L1 by glutamine deprivation in T24 cells was at 24 h, and the PD-L1 upregulation at 48 h was slightly lower than that at 24 h (Figure 1E). The protein levels of PD-L1 were elevated from 9 to 48 h in T24 cells (Figure 1G). The mRNA and protein level of PD-L1 were significantly upregulated after glutamine deprivation for 15 h in TCCSUP cells, and the high expression was decreased at 48 and 72 h, especially at 72 h (Figures 1F,H). This result indicates that the expression of PD-L1 can be upregulated under glutamine deprivation. The immunofluorescence results in T24 cells also confirmed that the expression of PD-L1 could be induced by glutamine deprivation (Supplementary Figures S1A,B). These results were confirmed in two other bladder cancer cell lines, 5,637 and UMUC-3, and the expression of PD-L1 was also upregulated by glutamine deprivation (Supplementary Figure S2).

### Upregulation of PD-L1 Induced by Glutamine Deprivation Through Activating the EGFR/MEK/ERK/C-Jun Signaling Pathway

**FIGURE 2:**
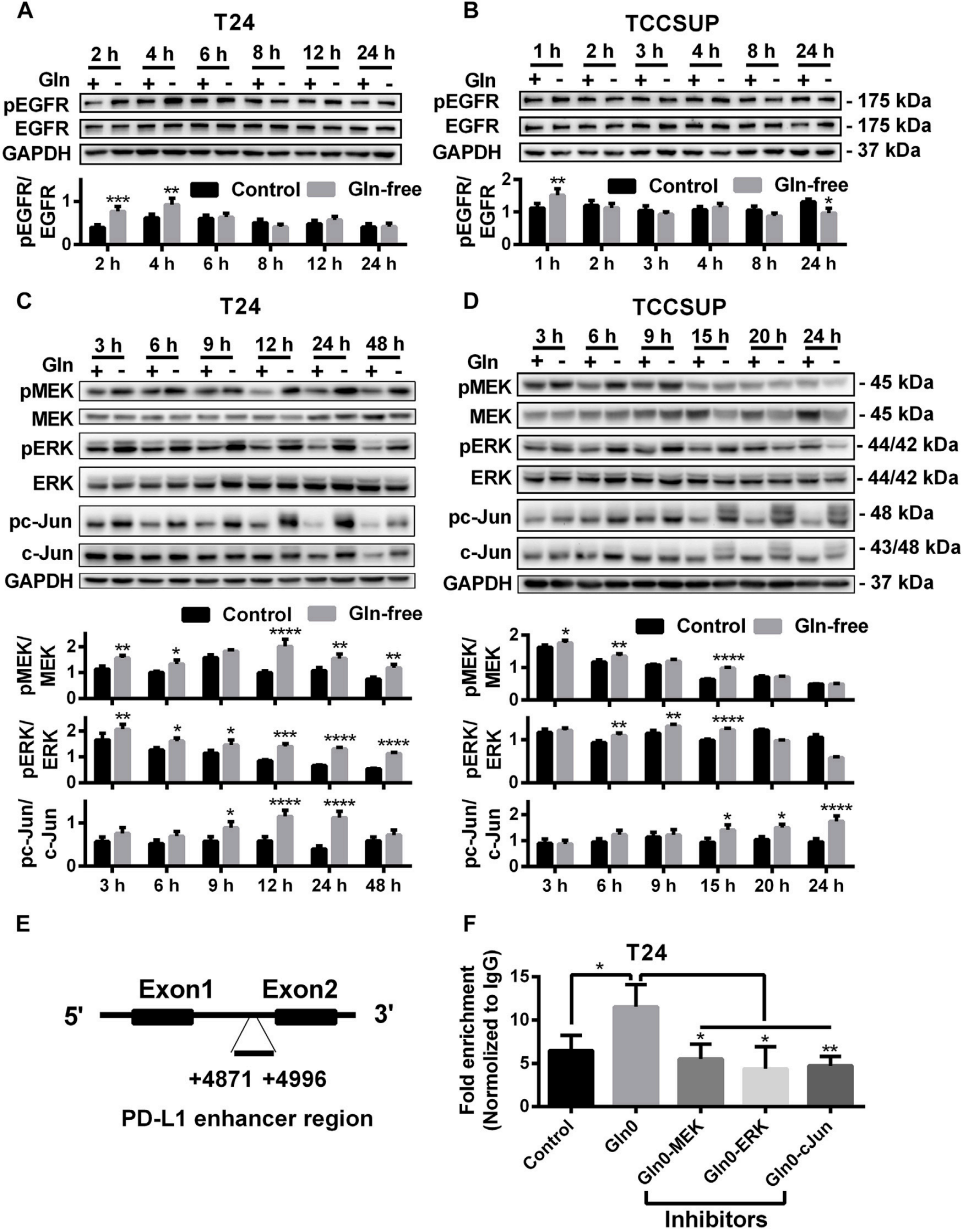
The protein levels of the EGFR/MEK/ERK/c-Jun pathway by glutamine deprivation. **(A)** The protein level of pEGFR in T24 cells after culturing in glutamine-free culture medium for 2–24 h (+: glutamine 300 mg/L; -: glutamine 0 mg/L). **(B)** The protein level of pEGFR in TCCSUP cells after culturing in glutamine-free culture medium for 1–24 h. **(C)** The protein levels of pMEK, pERK and pc-Jun in T24 cells after culturing in glutamine-free culture medium for 3–48 h. **(D)** The protein levels of pMEK, pERK and pc-Jun in TCCSUP cells after culturing in glutamine-free culture medium for 3–24 h. **(E)** The binding region of the AP-1 component c-Jun to the PD-L1 enhancer. **(F)** C-Jun enrichment at the PD-L1 gene enhancer in the control and glutamine deprivation with or without inhibitors was performed by ChIP coupled with qPCR. Ratio quantification of the specific phosphorylations was analysed. The results were considered significant at *p* < 0.05 (*p < 0.05, **p < 0.01, ***p < 0.001, ****p < 0.0001).

To investigate whether PD-L1 upregulation induced by glutamine deprivation was mediated through activation of the EGFR/MEK/ERK/c-Jun signaling pathway in T24 and TCCSUP cells, the protein expression of pEGFR, pMEK, pERK and pc-Jun after culturing in glutamine-free medium for different durations was determined by Western blot analysis. The expression of pEGFR was elevated at 2 and 4 h (Figure 2A), and the protein levels of pMEK and pERK in T24 cells were increased from 3 to 48 h, and pc-Jun was upregulated from 9 to 24 h (Figure 2C). The immunofluorescence results also confirmed that pMEK, pERK and pc-Jun levels could be increased by glutamine deprivation (Supplementary Figures S1C–H). The expression of pEGFR in TCCSUP cells was elevated at 1 h (Figure 2B). The levels of pMEK and pERK were upregulated from 6 to 15 h and pc-Jun was upregulated from 15 to 24 h (Figure 2D).

To investigate whether c-Jun, a downstream regulator in the MEK/ERK signaling pathway, regulates the transcription of PD-L1 under glutamine deprivation, we performed a ChIP assay to confirm the binding region of c-Jun to the PD-L1 gene in T24 cells. The results showed that there was significant binding of c-Jun to the PD-L1 enhancer region and that the fold enrichment was higher in the glutamine deprivation group than in the control group. In addition, the fold enrichment was reduced when inhibitors of the MEK/ERK/c-Jun signaling pathway (MEK inhibitor: U0126; ERK inhibitor: SCH772984; c-Jun inhibitor: SP600125) were added (Figures 2E,F). In summary, our results showed that glutamine deprivation may regulate the transcription of PD-L1 by activating the MEK/ERK/c-Jun pathway.

**FIGURE 3:**
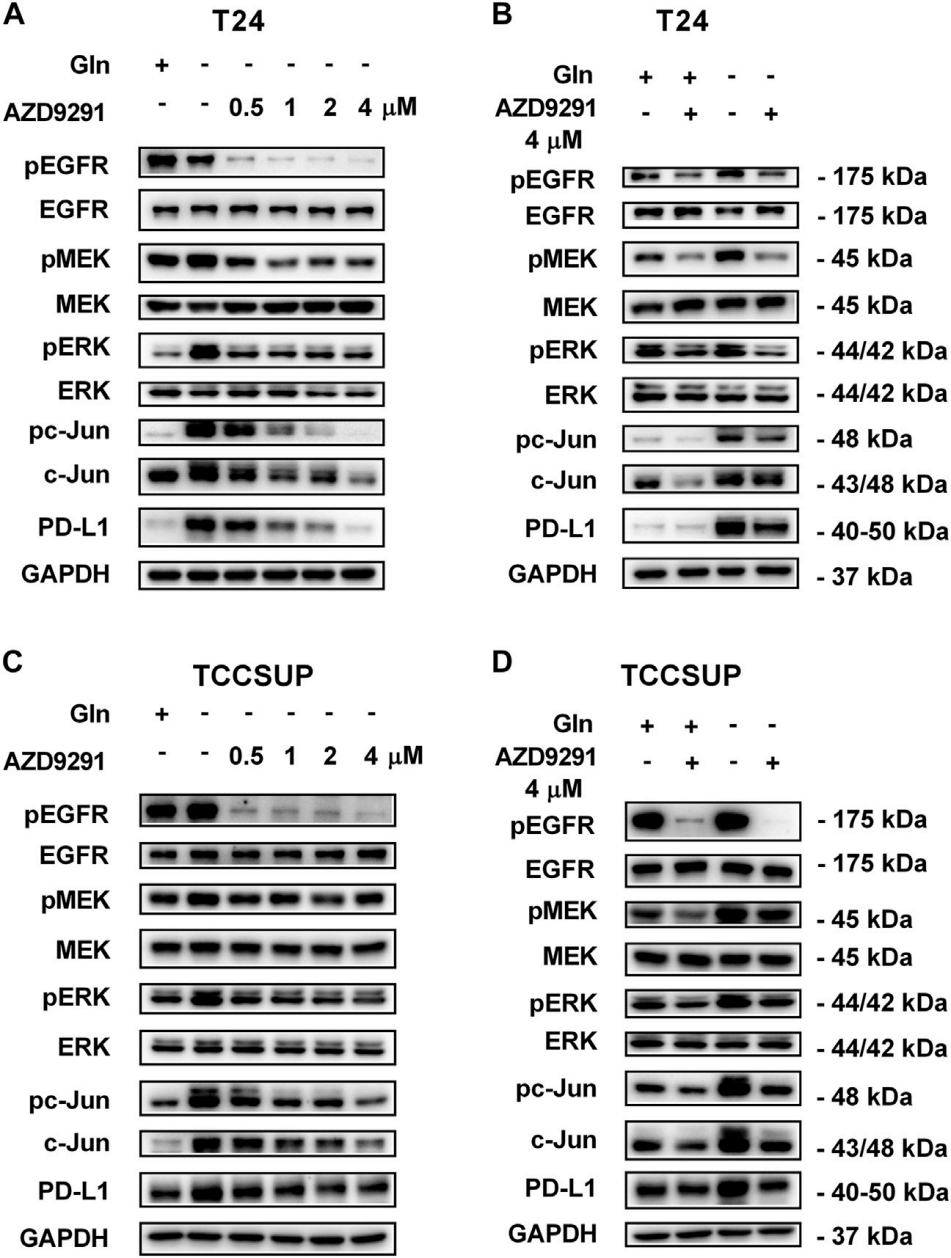
Inhibiting EGFR activation can reduce the elevated PD-L1 level induced by glutamine deprivation in T24 and TCCSUP cells. **(A)** The protein levels of pEGFR, pMEK, pERK, pc-Jun and PD-L1 in T24 cells cultured in glutamine-free medium with different concentrations of the pEGFR inhibitor (AZD9291: 0, 0.5, 1, 2 and 4 μM) for 24 h. **(B)** The protein levels of pEGFR, pMEK, pERK, pc-Jun and PD-L1 in T24 cells after culturing in normal or glutamine-free culture medium with AZD9291 (4 μM) for 24 h. **(C)** The protein levels of pEGFR, pMEK, pERK, pc-Jun and PD-L1 in TCCSUP cells cultured in glutamine-free medium with different concentrations of the pEGFR inhibitor (AZD9291: 0, 0.5, 1, 2 and 4 μM) for 15 h. **(D)** The protein levels of pEGFR, pMEK, pERK, pc-Jun and PD-L1 in TCCSUP cells after culturing in normal or glutamine-free culture medium with AZD9291 (4 μM) for 15 h.

**FIGURE 4:**
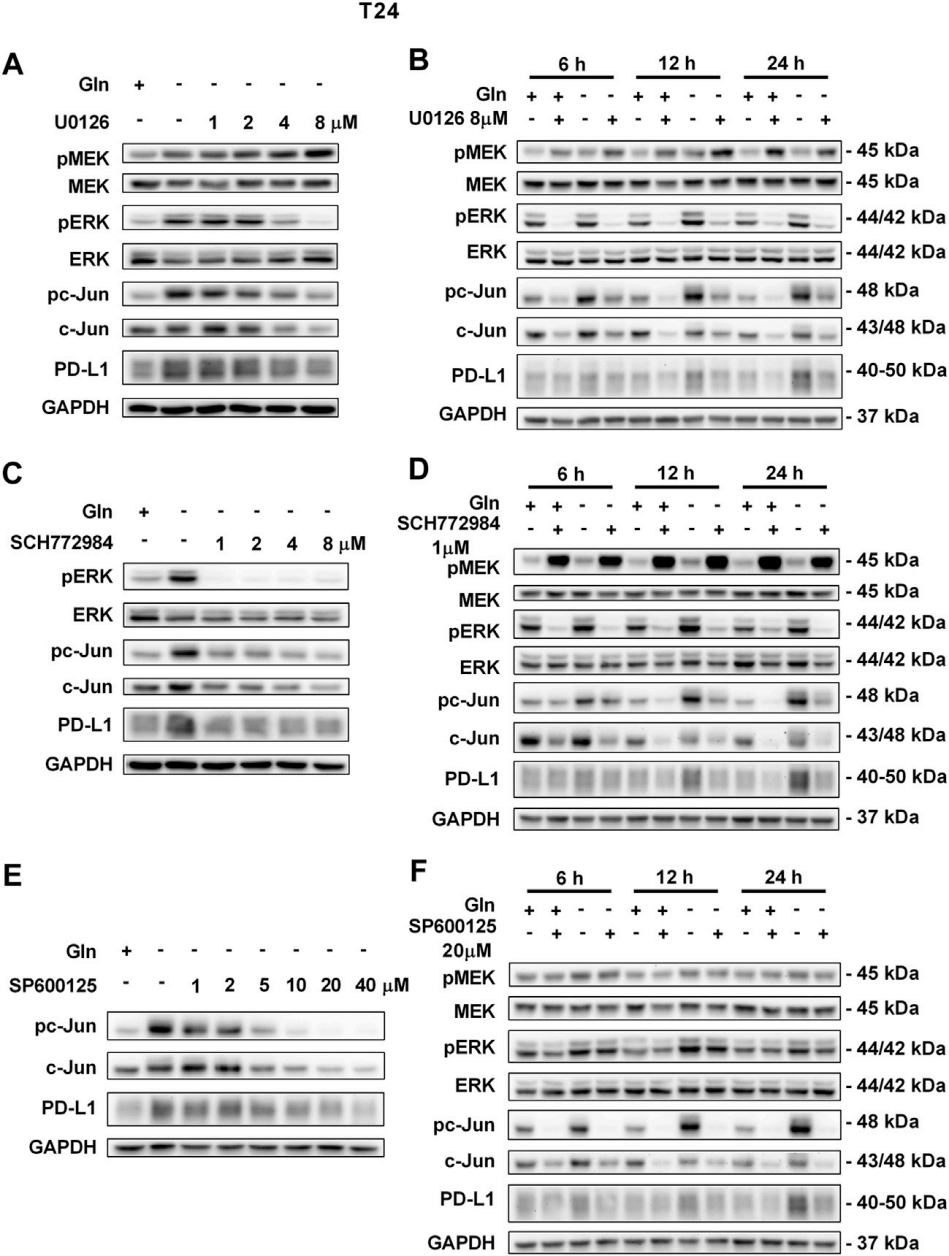
Inhibiting pMEK, pERK or pc-Jun activation can reduce the elevated PD-L1 level induced by glutamine deprivation in T24 cells.**(A)** The protein levels of pMEK, pERK, pc-Jun and PD-L1 in T24 cells cultured in glutamine-free medium with different concentrations of the pMEK inhibitor (U0126: 0, 1, 2, 4, and 8 μM) for 24 h. **(B)** The protein levels of pMEK, pERK, pc-Jun and PD-L1 in T24 cells after culturing in normal or glutamine-free culture medium with the pMEK inhibitor (8 μM) for 6, 12 and 24 h. **(C)** The protein levels of pERK, pc-Jun and PD-L1 in T24 cells cultured in glutamine-free medium with different concentrations of the pERK inhibitor (SCH772984: 0, 1, 2, 4, and 8 μM) for 24 h. **(D)** The protein levels of pMEK, pERK, pc-Jun and PD-L1 in T24 cells after culturing in normal or glutamine-free culture medium with the pERK inhibitor (1 μM) for 6, 12 and 24 h. **(E)** The protein levels of pc-Jun and PD-L1 in T24 cells cultured in glutamine-free medium with different concentrations of the pc-Jun inhibitor (SP600125: 0, 1, 2, 5, 10, 20, and 40 μM) for 24 h. **(F)** The protein levels of pMEK, pERK, pc-Jun and PD-L1 in T24 cells after culturing in normal or glutamine-free culture medium with the pc-Jun inhibitor (20 μM) for 6, 12 and 24 h.

**FIGURE 5:**
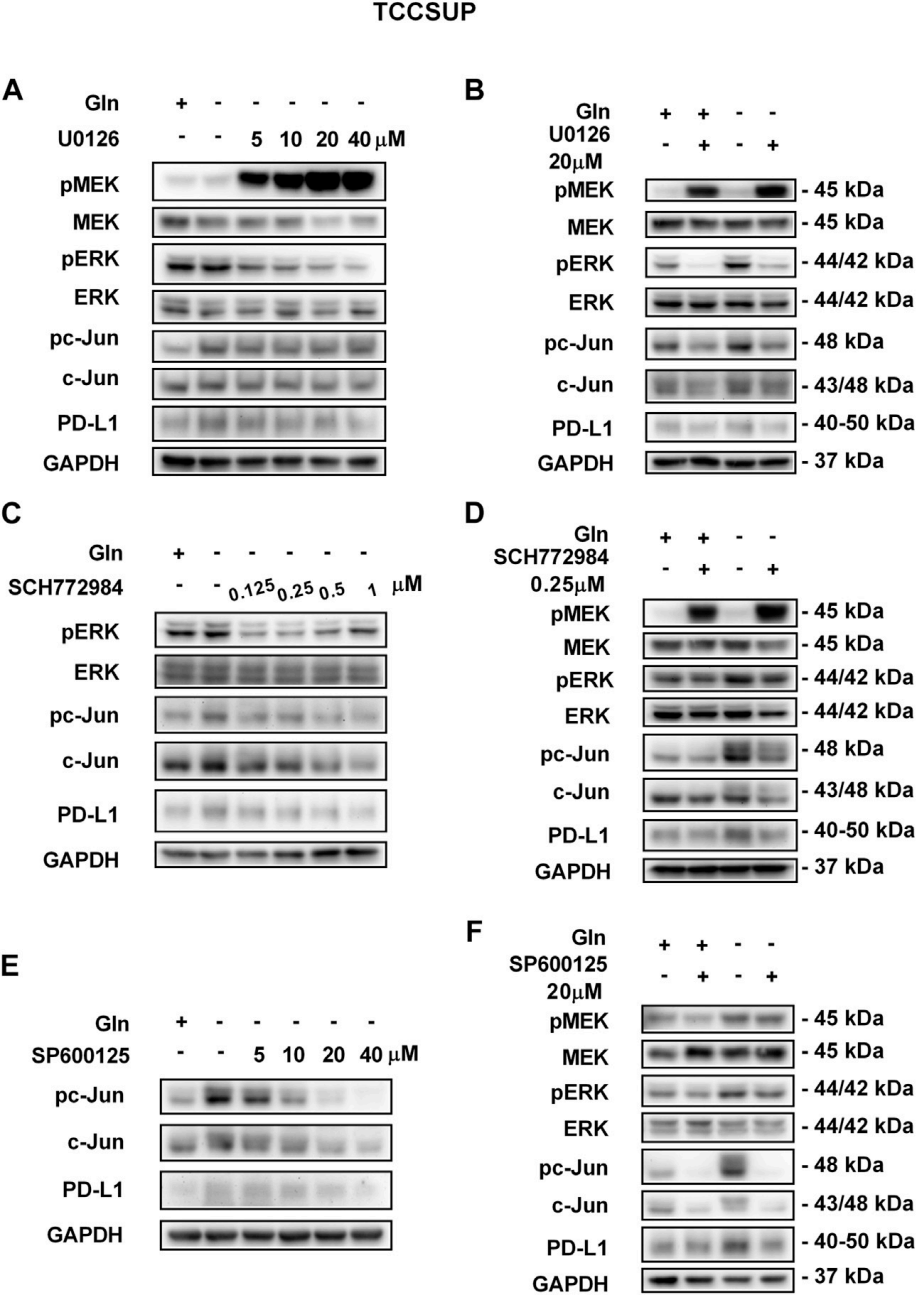
Inhibiting pMEK, pERK or pc-Jun activation can reduce the elevated PD-L1 level induced by glutamine deprivation in TCCSUP cells. **(A)** The protein levels of pMEK, pERK, pc-Jun and PD-L1 in TCCSUP cells after culturing in glutamine-free culture medium with different concentrations of the pMEK inhibitor (U0126: 0, 5, 10, 20, and 40 μM) for 15 h. **(B)** The protein levels of pMEK, pERK, pc-Jun and PD-L1 in TCCSUP cells after culturing in normal or glutamine-free culture medium with the pMEK inhibitor (20 μM) for 15 h. **(C)** The protein levels of pERK, pc-Jun and PD-L1 in TCCSUP cells after culturing in glutamine-free culture medium with different concentrations of the pERK inhibitor (SCH772984: 0, 0.125, 0.25, 0.5, 1 μM) for 15 h. **(D)** The protein levels of pMEK, pERK, pc-Jun and PD-L1 in TCCSUP cells after culturing in normal or glutamine-free culture medium with the pERK inhibitor (0.25 μM) for 15 h **(E)** The protein expression of pc-Jun and PD-L1 in TCCSUP cells after culturing in glutamine-free culture medium with different concentrations of the pc-Jun inhibitor (SP600125: 0, 5, 10, 20, and 40 μM) for 15 h. **(F)** The protein levels of pMEK, pERK, pc-Jun and PD-L1 in TCCSUP cells after culturing in normal or glutamine-free culture medium with the pc-Jun inhibitor (20 μM) for 15 h.

**FIGURE 6:**
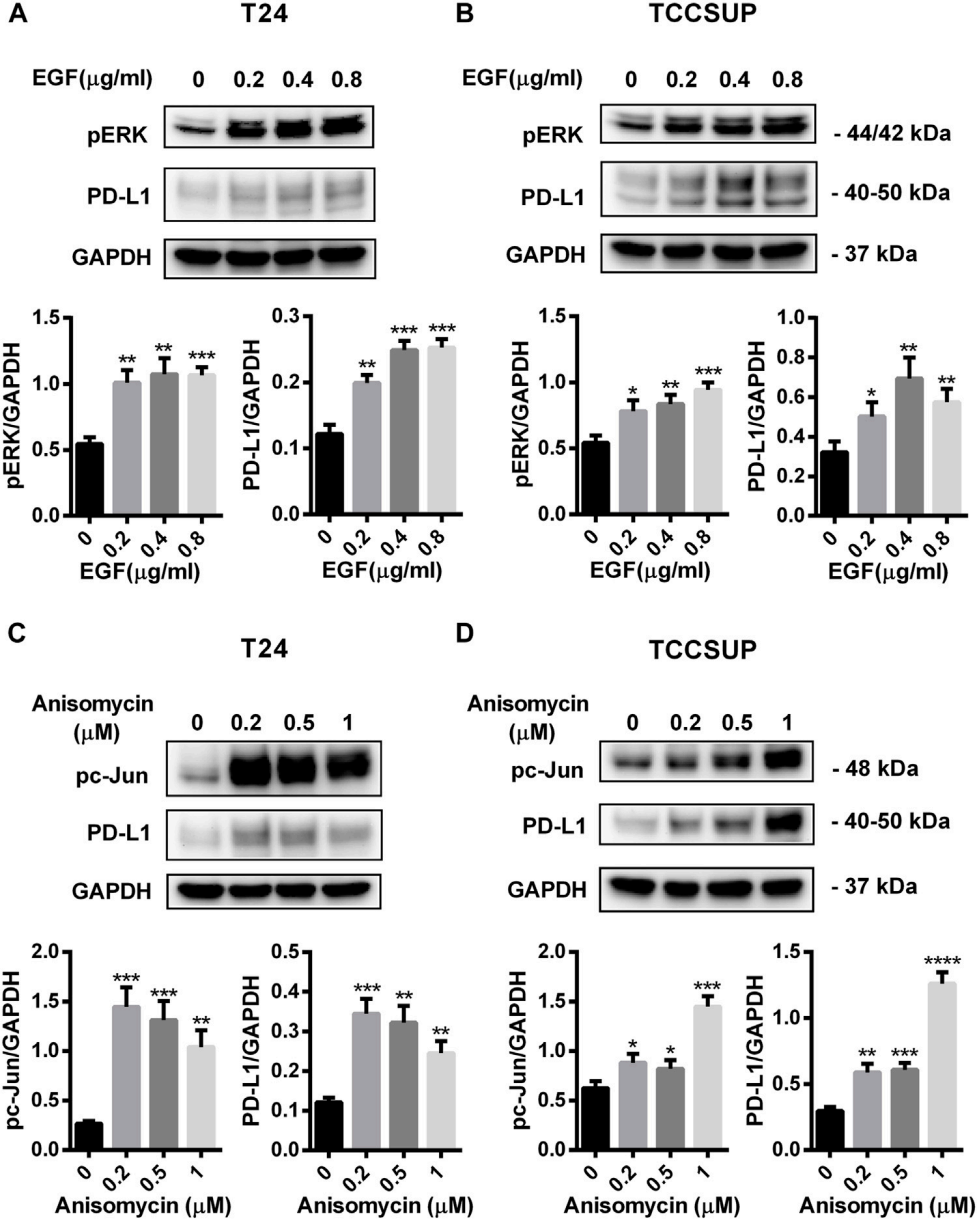
PD-L1 expression was upregulated by EGF and Anisomycin in T24 and TCCSUP cells. **(A,B)** The protein levels of pERK and PD-L1 in T24 and TCCSUP cells after culturing with different concentrations of EGF (a ligand of EGFR, often used to activate ERK signal, 0, 0.2, 0.4 and 0.8 μg/ml) for 2 h **(C,D)** The protein levels of pc-Jun and PD-L1 in T24 and TCCSUP cells after culturing with different concentrations of Anisomycin (a JNK activator, which can activate c-Jun, 0, 0.2, 0.5 and 1 μM) for 24 h. The densitometric analysis of proteins was performed and the results were normalized to GAPDH. The results were considered significant at *p* < 0.05 (*p < 0.05, **p < 0.01, ***p < 0.001, ****p < 0.0001).

To further confirm that PD-L1 upregulation is induced by glutamine deprivation through EGFR/MEK/ERK/c-Jun signaling pathway activation, inhibitors of EGFR, MEK, ERK and c-Jun were used in T24 and TCCSUP cells. First, cells cultured in glutamine-free culture medium were simultaneously treated with different concentrations of EGFR, MEK, ERK and c-Jun inhibitors to determine the optimal dosages. Then, the cells were cultured in normal medium and glutamine-free culture medium with or without the optimal dose of EGFR, MEK, ERK or c-Jun inhibitors. The results showed that pEGFR, pMEK, pERK, pc-Jun and PD-L1 levels were all reduced by the EGFR inhibitor (AZD9291) and the optimal doses were 4 μM in the two cell lines (Figures 3A,C). Upon the addition of the optimal doses of AZD9291, pEGFR, pMEK, pERK , pc-Jun and PD-L1 were all reduced no matter the glutamine present or not (Figures 3B,D). The corresponding protein quantification analysis of Figure 3 was shown in Supplementary Figure S3. When the dose of the pMEK inhibitor (U0126) increased, the pMEK level was elevated by negative feedback (Eppstein et al., 2006), but the subsequent pERK, pc-Jun and PD-L1 levels were significantly reduced. The optimal doses of U0126 in T24 and TCCSUP cells were 8 and 20 μM respectively (Figures 4A, 5A). The optimal doses of inhibitors for pERK and pc-Jun in T24 cells were 1 μM (SCH772984) and 20 μM (SP600125), respectively (Figures 4C,E). In TCCSUP cells, they were 0.25 and 20 μM, respectively (Figures 5C,E). The difference in dose is probably due to different sensitivities to inhibitors in different cells. Upon the addition of the optimal doses of pMEK and pERK inhibitors to T24 and TCCSUP cells, the levels of pERK, pc-Jun and PD-L1 were reduced in both normal and glutamine-free culture medium. Furthermore, inhibiting pMEK or pERK activation at T24 reduced the elevated PD-L1 expression induced by glutamine deprivation at 6, 12, and 24 h, especially at 12 and 24 h (Figures 4B,D), and inhibiting pMEK or pERK activation at TCCSUP also reduced the elevated PD-L1 expression induced by glutamine deprivation at 15 h (Figures 5B,D). Upon the addition of the optimal dose (20 μM) of pc-Jun inhibitor to T24 and TCCSUP cells, pc-Jun and PD-L1 were reduced in both normal and glutamine-free culture medium. These results showed that inhibiting pc-Jun activation could decrease the elevated PD-L1 level induced by glutamine deprivation (Figures 4F, 5F). The protein quantification analyses of Figures 4, 5 were shown in Supplementary Figures S4, S5 respectively (Supplementary Figures S4, S5). Except for the inhibitors, positive controls were performed by EGF (a ligand of EGFR, often used to activate ERK signaling) and anisomycin (a JNK activator, which can activate c-Jun). The results showed that pERK and PD-L1 levels could be elevated by EGF in both T24 and TCCSUP cells, and this effect increases with EGF dose increasing (Figures 6A,B). The pc-Jun and PD-L1 levels were also elevated by anisomycin in the two cell lines (Figures 6C,D). Overall, our results clearly showed that inhibiting EGFR/MEK/ERK/c-Jun signaling pathway activation could reduce the elevated PD-L1 expression induced by glutamine deprivation.

### Reducing the Upregulated PD-L1 and MEK/ERK/C-Jun Signaling Pathway Through Glutamine Recovery *in vitro* and *in vivo*

**FIGURE 7:**
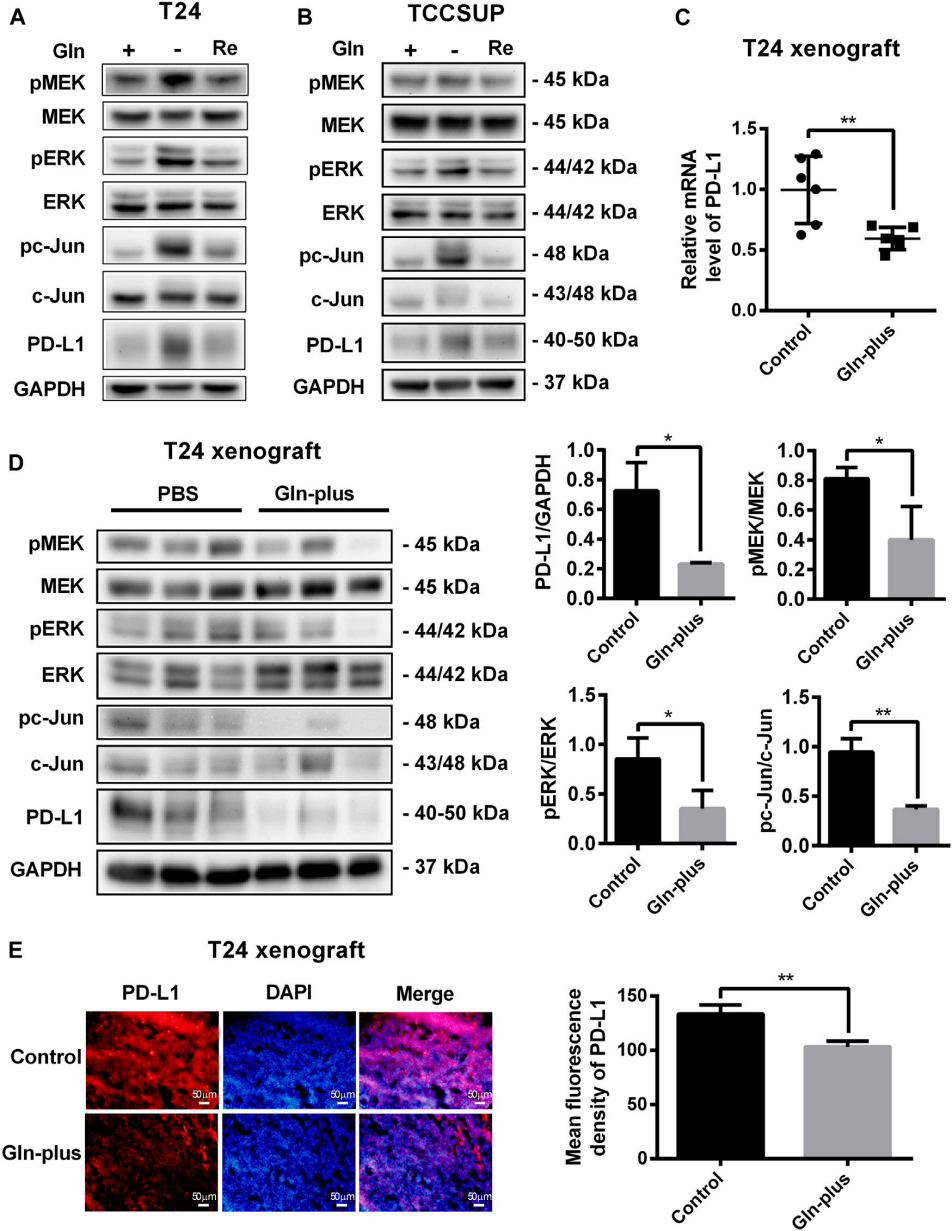
Upregulation of PD-L1 and the MEK/ERK/c-Jun pathway were reduced after glutamine recovery *in vitro* and *in vivo*. **(A,B)** The protein levels of pMEK, pERK, pc-Jun and PD-L1 in T24 and TCCSUP cells after glutamine recovery (Re: glutamine recovery). **(C)** The mRNA level of PD-L1 after glutamine supplementation in T24 xenografts. **(D)** The protein levels of pMEK, pERK, pc-Jun and PD-L1 after glutamine supplementation in T24 xenografts. Three samples for each group were shown. The densitometric analysis of proteins was performed. **(E)** PD-L1 expression after glutamine supplementation in T24 xenografts was investigated by fluorescence microscopy and the mean fluorescence density was analyzed by ImageJ software. The results were considered significant at *p* < 0.05 (*p < 0.05, **p < 0.01).

To investigate the changes in PD-L1 expression and MEK/ERK/c-Jun signaling pathway activation after glutamine recovery, T24 and TCCSUP cells were cultured in glutamine-free culture medium for 24 or 15 h; the culture medium was then replaced with normal medium for another 24 h. The expression of PD-L1, pMEK, pERK and pc-Jun was detected by Western blotting. The results showed that the elevated PD-L1 level induced by glutamine deprivation was decreased by glutamine recovery and that MEK/ERK/c-Jun pathway activation was also decreased after glutamine recovery (Figures 7A,B). The protein quantification analyses of Figures 7A,B were shown in Supplementary Figure S6.

To further confirm whether this PD-L1 recovery occurred in tumor tissues that lacked glutamine, an *in vivo* xenograft model was used. The mice bearing tumors were arbitrarily assigned to two groups, namely, the PBS control group and glutamine complement group, to receive intratumoral injections. After six treatments, no significant abnormalities were observed in the mice, and there were no abnormal changes in the body weights of either the control or experimental group. The tumors were harvested to detect PD-L1 expression and MEK/ERK/c-Jun pathway activation. Our results showed that the mRNA level of PD-L1 in the glutamine supplementation group was obviously lower than that in the control group (Figure 7C), which was consistent with the immunofluorescence results (Figure 7E). The results of the Western blot analysis also confirmed that PD-L1 expression could be reduced by glutamine supplementation in T24 xenografts (Figure 7D). Furthermore, we confirmed that MEK/ERK/c-Jun pathway activation was also reduced by glutamine supplementation (Figure 7D).

### PD-L1 Upregulation by Glutamine Deprivation Influences T Cell Function

**FIGURE 8:**
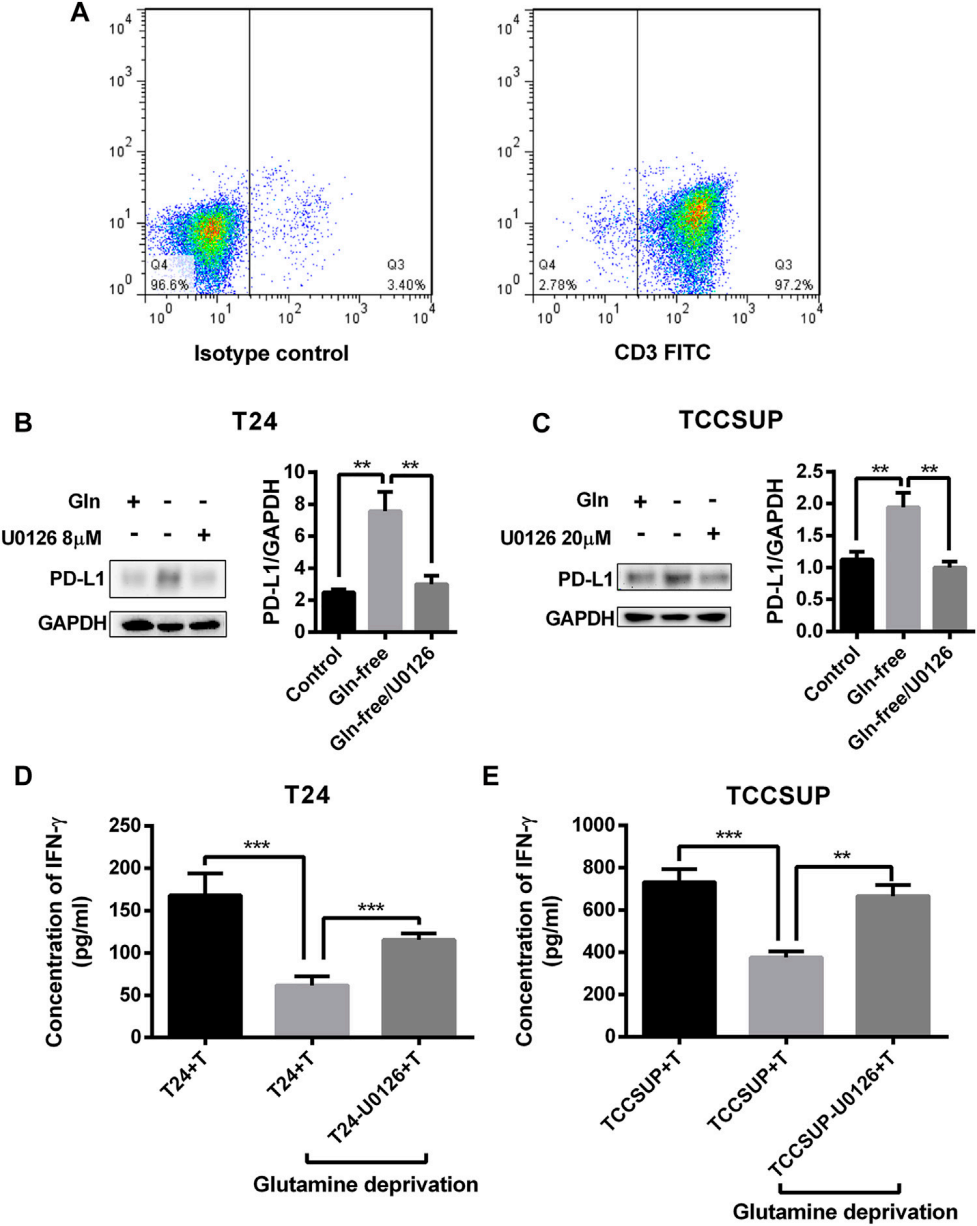
PD-L1 upregulation by glutamine deprivation in T24 and TCCSUP cells decreases IFN-γ production by T cells in coculture conditions. **(A)** The purity of CD3^+^ T cells was assessed by flow cytometry. **(B)** The protein levels of PD-L1 in T24 cells after culturing in normal medium and glutamine-free culture medium with or without U0126 (8 μM). **(C)** The protein expression of PD-L1 in TCCSUP cells cultured in normal medium and glutamine-free culture medium with or without U0126 (20 μM). **(D)** The production of IFN-γ by T cells after coculturing with T24 cells by different pretreatments. **(E)** The production of IFN-γ by T cells after coculturing with TCCSUP cells by different pretreatments. The densitometric analysis of proteins was performed and the results were normalized to GAPDH. The results were considered significant at *p* < 0.05 (**p < 0.01, ***p < 0.001).

To investigate whether PD-L1 upregulation by glutamine deprivation in T24 and TCCSUP cells affects the function of T cells, T24 and TCCSUP cells were pretreated under glutamine deprivation conditions with pMEK inhibitor or not. Then, CD3^+^ T cells were cocultured with bladder cancer cells pretreated with different conditions, and IFN-γ production was detected by ELISA. The results of flow cytometry showed that the purity of CD3^+^ T cells was greater than 90% after culturing (Figure 8A). The expression of PD-L1 in T24 and TCCSUP cells was elevated by glutamine deprivation and decreased when U0126 was added simultaneously (Figures 8B,C). Afterward, T cells were cultured with T24 or TCCSUP cells with different pretreatments for 15 h in normal medium. Even though the elevated PD-L1 could decreased slightly due to glutamine recovery in 15 h, it was still higher than the normal control (Supplementary Figure S7). Compared with the normal control group, the IFN-γ concentration was decreased after culturing with glutamine-free-treated T24 and TCCSUP cells but was enhanced after culturing with glutamine-free/U0126-treated cells (Figures 8D,E). Overall, our results suggested that PD-L1 upregulation by glutamine deprivation decreased IFN-γ production by T cells, and that the MEK inhibitor reversed this process.

## Discussion

The expression of PD-L1 in the tumor microenvironment is involved not only in signaling pathways, transcriptional regulation and epigenetic regulation but also in inflammatory cytokine production (Boussiotis 2016; Chen et al., 2016). However, few studies have reported whether nutrients in the tumor microenvironment regulate PD-L1 expression in cancer cells. Glutamine metabolism inherent in tumors is well studied, but its effects on the immune microenvironment are not well studied (Faiena et al., 2019). Because of the lack of glutamine in tumor tissues, we assessed whether glutamine deprivation was associated with PD-L1 expression and further influenced immune function. The majority of bladder cancer cases are relevant to transitional cell carcinoma. The T24 cell line, which is derived from human bladder transitional cell carcinoma, is a representative malignant bladder cancer cell line progressing to the muscle layer of the bladder wall, and our previous study revealed that the T24 cell line had a higher rate of tumorigenesis in nude mice. In our study, PD-L1 expression in T24 cells was upregulated by glutamine deprivation. We further found that glutamine deprivation could activate the EGFR/MEK/ERK/c-Jun signaling pathway, which mediated the upregulation of PD-L1 expression. Furthermore, PD-L1 upregulation and MEK/ERK/c-Jun pathway activation were reduced after glutamine recovery *in vitro* and *in vivo*. Additionally, a preliminary study in T24 cells suggested that the PD-L1 elevation induced by glutamine deprivation inhibited the function of T cells. In addition, we performed key *in vitro* experiments and coculture assays using another bladder cancer cell line (TCCSUP, which is derived from anaplastic transitional cell carcinoma) to further confirm that the expression of PD-L1 was upregulated by glutamine deprivation through the EGFR/MEK/ERK/c-Jun pathway to impair T cell function.

Glutamine, a nonessential amino acid, is a major carbon and nitrogen source that supports cancer proliferation and survival by replenishing tricarboxylic acid (TCA) cycle intermediates (Boroughs and DeBerardinis 2015; Coloff et al., 2016). However, in the tumor microenvironment, glutamine is depleted by increased glutamine uptake and poor vascularization. In contrast to other amino acids, glutamine is most depleted in the core regions of solid tumors, including melanoma and pancreatic adenocarcinoma (Kamphorst et al., 2015; Pan et al., 2016). Therefore, cancer cells often develop strategies to survive during periods of glutamine starvation. Studies have demonstrated that the phosphatase PP2A and the transcription factor p53 can promote cancer cell adaptation to glutamine deprivation in colon cancer line (Reid et al., 2013; Tajan et al., 2018) as well as other mechanisms (Pan et al., 2016; Takeuchi et al., 2018). However, in T24 and TCCSUP cells, the mRNA levels of p53 were not significantly different between the control group and glutamine-free group (Supplementary Figure S8). In this study, we discovered that PD-L1 expression in bladder cancer cells was upregulated by glutamine deprivation. This is a newly discovered regulatory mechanism involved in PD-L1 expression in bladder cancer cells and may be one of the strategies used by cancer cells to adapt to nutrient-limiting conditions by immune escape. However, glutamine deprivation-mediated PD-L1 upregulation at 48 h was lower than that at 24 h. This result suggested that glutamine depletion could temporarily upregulate PD-L1 expression to help cancer cells survive in adverse environments. However, because glutamine is essential for the biosynthesis of amino acids, PD-L1 upregulation is weakened when cells suffer from glutamine deprivation over the long term. Similarly, although cancer cells have been reported to develop strategies to support proliferation while glutamine is nearly depleted from culture medium, the cell proliferation rates are markedly reduced when cells are cultured in glutamine-free culture medium (Takeuchi et al., 2018).

It has been reported that pEGFR can be activated by glutamine deprivation in pancreatic ductal adenocarcinoma (PDAC) tumors (Lee et al., 2019; Lee and Commisso 2020). Other studies have shown that ASCT2 (SLC1A5), a glutamine transporter, is an EGFR-associated protein in EGFR-overexpressing human head and neck squamous cell carcinoma (HNSCC) (Lu et al., 2016). The MEK/ERK pathway is an oncogenic signaling pathway that is well known for its induction of PD-L1 expression (Ota et al., 2015; Kumar and Sharawat 2018). Recent studies have demonstrated that cancer cells under extreme conditions can upregulate PD-L1 expression through the MEK/ERK pathway. In lung cancer cells, PD-L1 levels can be altered by radiation via the IL-6-MEK/ERK signaling pathway, which can help cancer cells escape killing by natural killer (NK) cells (Shen et al., 2017). In esophageal squamous cell carcinoma, PD-L1 expression can also be increased by chemotherapeutic treatments through activating the EGFR/ERK signaling pathway (Ng et al., 2018). In this study, PD-L1 expression was upregulated by glutamine deprivation through activating the EGFR/MEK/ERK pathway in T24 and TCCSUP cells. pEGFR was activated very quickly by glutamine deprivation at 2 or 1 h in T24 and TCCSUP cells respectively. However, according to our previous study in renal cancer cells, pEGFR was activated by glutamine deprivation at 8 h (Ma et al., 2020). This result demonstrates that the EGFR signaling pathway in different cancer cell lines maybe has different sensitivities to glutamine deprivation. Inhibiting pEGFR, pMEK or pERK activation could decrease the PD-L1 elevation induced by glutamine deprivation. However, the phosphorylation time points of pMEK and pERK in T24 and TCCSUP cells were different, and the two cell lines had different sensitivities to pMEK and pERK inhibitors. In addition, pc-Jun, a transcription factor that is downstream of ERK, can regulate PD-L1 expression in NSCLC and melanoma cells (Jiang et al., 2013; Chen et al., 2015). In our study, the level of pc-Jun could be activated by glutamine deprivation, and ChIP analysis confirmed that c-Jun had significant binding to the enhancer region of PD-L1, which regulates the transcription of PD-L1 in T24 cells. Inhibiting pc-Jun activation could reduce PD-L1 elevation. Consequently, PD-L1 upregulation induced by glutamine deprivation was mediated through activation of the EGFR/MEK/ERK/c-Jun signaling pathway. Furthermore, the elevated PD-L1 level induced by glutamine deprivation could be decreased by glutamine recovery, and the activation of the MEK/ERK/c-Jun pathway was also decreased after glutamine recovery. However, the regulatory mechanism in detail involved in this pathway is still unknown. It was reported that Ca^2+^ is associated with EGFR in NSCLC and HNSCC cells (Wang et al., 2017; Kim et al., 2020), and an increase in intracellular Ca^2+^ levels could activate the ERK pathway (Ayush et al., 2016; Wang et al., 2019). We preliminarily found that glutamine deprivation could increase intracellular Ca^2+^ levels in T24 cells. This result suggested that Ca^2+^ flux may involved in the activation of EGFR/MEK/ERK/c-Jun signaling pathway by glutamine deprivation. However, this hypothesis needs to be fully confirmed through further investigations. Glutamine levels have been demonstrated to be lower in numerous tumor tissues than in normal tissues (Roberts and Frankel 1949). Moreover, in an *in vivo* xenograft nude mouse model, glutamine was reported to be even lower in the core regions of tumors than in the periphery (Reid et al., 2013). In this study, we also used an *in vivo* xenograft model to confirm that supplementation with glutamine could reduce PD-L1 expression in glutamine-depleted tumor tissues.

We speculated that upregulated PD-L1 expression may be one of the strategies that cancer cells use to adapt to glutamine deprivation by immune escape, so we performed a preliminary study on the impacts of PD-L1 on T cells. Our results suggest that PD-L1 upregulation induced by glutamine deprivation could reduce IFN-γ production by T cells and that treatment with the pMEK inhibitor could recover the capacity of T cells to produce IFN-γ. Therefore, we suggest that PD-L1 upregulation under glutamine deprivation in bladder cancer cells is a strategy for inhibiting T cell function under extreme nutritional conditions.

This study reveals a new regulatory mechanism for PD-L1 overexpression that involves glutamine deficiency in bladder cancer cells and the signaling pathways involved in this regulatory process. The results are helpful for understanding how cancer cells promote survival in a glutamine-poor environment. Notably, this study examined whether glutamine deprivation could upregulate PD-L1 expression by activating the EGFR/MEK/ERK/c-Jun signaling pathway and performed a preliminary investigation of its effect on the immune system. However, the regulatory mechanism in detail and the complex regulatory relationships with the immune system remain unknown. Further *in situ* studies with animal models and clinical specimen analyses of bladder cancer also need to be performed. These problems need to be fully understood to increase the efficacy of targeted therapy and will be the focus of our future investigations.

## Conclusion

In this study, we observed that PD-L1 expression could be elevated by glutamine deprivation by activating the EGFR/MEK/ERK/c-Jun pathway and that this process could reduce the production of IFN-γ by T cells. Moreover, the elevation of PD-L1 expression and the activation of the MEK/ERK/c-Jun pathway could be decreased by glutamine recovery, which was confirmed in a xenograft model *in vivo*. Our findings provide a new mechanism by which to regulate PD-L1 expression in bladder cancer cells that may be a strategy for cancer cell survival under glutamine deprivation conditions and for escape from the immune system.

## Materials and Methods

### Cell Culture and Glutamine Deprivation

The T24 cell line (RRID: CVCL_0554) was purchased from the Typical Culture Preservation Commission Cell Bank (Shanghai, China) and the TCCSUP cell line (RRID: CVCL_1738) was purchased from Procell (Wuhan, China). All human cell lines were authenticated using STR profiling within the last 3 years, and no mycoplasma contamination was identified. The cells (1 × 10^6^ cells/well) were seeded in 24-well plates at 37°C and 5% CO_2_. After 24 h, the culture medium was replaced with low-glutamine or glutamine-free medium. Cells cultured in normal culture medium served as controls. The expression of pMEK, pERK, pc-Jun and PD-L1 was determined by qPCR, Western blot analysis or immunofluorescence. The enrichment of c-Jun at the PD-L1 gene was performed by ChIP. For the glutamine recovery experiment, normal medium was replaced after 24 h of glutamine deprivation in T24 cells and after 15 h of glutamine deprivation in TCCSUP.

### Assays of Inhibitor Treatment

To investigate the signaling pathways related to PD-L1 upregulation under glutamine deprivation, inhibitors were used to suppress the EGFR/MEK/ERK/c-Jun signaling pathway. EGFR inhibitor (AZD-9291, cat. no HY-15772), MEK1/2 inhibitor (U0126, cat. no HY-12031), ERK1/2 inhibitor (SCH772984, cat. no HY-50846), JNK1/2/3 inhibitor (SP600125, cat. no HY-12041) and JNK activator (Anisomycin, cat. no HY-18982) were obtained from MCE (MedChemExpress, Monmouth Junction, NJ, United States). Recombinant human EGF was obtained from Novus (St. Louis, Missouri, United States). Then, cells were harvested for Western blot analysis or ChIP assays.

### Glutamine Supplementation Assay in an *in vivo* Xenograft Model

Male nude mice (BALB/c; 4–5 weeks old) were obtained from the Beijing Charles River Laboratory Animal Technology (Beijing, China) and maintained under specific pathogen-free (SPF) conditions. Mice were injected with T24 cells (7 × 10^6^) on their backs. After several weeks, the tumors reached a size of approximately 5 × 5 mm^2^. Then twelve mice were randomly assigned to 2 groups, namely, the phosphate-buffered saline (PBS) control group and the glutamine supplementation group, to receive intratumoral injections. PBS and glutamine prepared in PBS (1.5 g/L, pH 7.4) were administered separately into the tumors at two locations (20 μl/location). Mice received anintratumoral injection every 3 days. After six injections, all the mice were sacrificed by dislocation of the cervical vertebra. The tumor tissue of each animal was excised and divided into three parts for different analyses: one for RT-PCR analysis, one for Western blot analysis, and one for immunofluorescence analysis. For the animal experiments, protocols were approved by the Institutional Animal Care and Use Committee of Affiliated Hospital of Qingdao University.

### IFN-γ Analysis of T Cells

Human peripheral T cells were obtained as previously described (Wang et al., 2018). The study was conducted with approval from the Institutional Review Board of the Affiliated Hospital of Qingdao University and written informed consent was obtained from the healthy volunteers. T24 and TCCSUP cells (5 × 10^5^ cells/well) were seeded in 24-well plates for 24 h, and then cultured in glutamine-free culture medium with 8 or 20 μM U0126 for another 24 h. Cells cultured in glutamine-free or normal culture medium were used as controls. Then, the culture media of all groups were replaced with fresh normal medium, containing 3 × 10^5^ T cells/well for direct contact with bladder cancer cells. After 15 h, PMA/Iono was added to stimulate T cells for 5 h. Then, the IFN-γ levels in the supernatants of the T cell cocultures were measured using a human IFN-γ ELISA kit (Elabscience, Wuhan, China).

### Western Blot Analysis

Total protein was extracted from cells or xenografts in SDS buffer, and a bicinchoninic acid (BCA) kit (Thermo Fisher Scientific, Waltham, MA, United States) was used to measure the protein concentration. After separation by SDS-polyacrylamide gel electrophoresis (PAGE), the proteins were transferred to PVDF membranes (Millipore, Billerica, United States). Then membranes were blocked in 5% fat-free milk in PBS-T for 1 h and incubated with primary antibody at 4°C overnight. The second day, the membranes were incubated with HRP-conjugated secondary antibodies (JacksonImmunoResearch, West Grove, PA, United States; the dilution was 1:10,000) at room temperature for 1 h and detected with an enhanced chemiluminescence kit (Millipore). AlphaView SA software was used for densitometric analysis, and the results were normalized to GAPDH to correct the differences in protein loading. The following anti-human primary antibodies were used: PD-L1 (#13684, Cell Signaling Technology, Beverly, MA, United States), phosphor-EGFR (#3777, CST), EGF receptor (#4267, CST), phospho-MEK1/2 (Ser217/221, #9154, CST), MEK1/2 (#8727, CST), phospho-ERK1/2 (Thr202/Tyr204, #4370, CST), ERK1/2 (#4695, CST), phospho-c-Jun (Ser73, #3270, CST), c-Jun (#9165, CST), and GAPDH (#G9545, Sigma-Aldrich, St. Louis, MO, United States).

### Immunofluorescence

T24 cells were grown on glass slides (WHB SCIENTIFIC, Shanghai, China) in 24-well culture plates and then treated with glutamine-free medium. First, the cells were fixed with 4% PBS-buffered paraformaldehyde for 15 min and washed with PBS several times. After blocking with 5% bovine serum albumin (BSA) for 30 min, the cells were incubated with primary antibodies against PD-L1, pMEK, pERK and pc-Jun for 1 h at room temperature. Then, the cells were counterstained with Cy3-conjugated secondary antibody (Jackson ImmunoResearch) in the dark for 1 h and the cell nuclei were stained with DAPI. Immunofluorescence images were obtained using fluorescence microscopy (Olympus, IX71, Tokyo, Japan) and confocal laser scanning microscopy (SPE; Leica, Heidelberg, Germany). In addition, four T24 xenografts each group embedded in optimal cutting temperature compound (OCT, Miles Inc., Elkhart, IN, United States) were sectioned into 5-μm-thick sections by a cryostat (SLEE International, Inc., New York, NY, United States). Then two slices for each xenografts were adhered to slides, fixed with 4% paraformaldehyde for 10 min and washed with PBS three times. After the xenograft sections were blocked for 1 h, they were incubated with PD-L1 antibody for 1 h at room temperature. The following steps were the same as those in the cell immunofluorescence method described above, and 3 fields were captured for each slice by using fluorescence microscopy.

### Quantitative Real-Time PCR

Total RNA from cells and xenografts was isolated by TRIzol (Code#9109, Takara, Kusatsu, Japan), and 500 ng of total RNA was then transcribed to cDNA by a PrimeScript™ RT reagent kit (Takara, Code#RR037A). RT-qPCR was performed with SYBR Green PCR master mix (Takara) by using a Roche LightCycler 480II real-time PCR instrument (Roche, Basel, Switzerland). The primers were as follows: PD-L1 (forward): 5′-TAT​GGT​GGT​GCC​GAC​TAC​AA-3’; PD-L1 (reverse): 5′-TGC​TTG​TCC​AGA​TGA​CTT​CG-3’; GAPDH (forward): 5′-CAG​GGC​TGC​TTT​TAA​CTC​TGG​TA-3’; and GAPDH (reverse): 5′-CAT​GGG​TGG​AAT​CAT​ATT​GGA​AC-3’. The fold changes in the expression of each gene were calculated using the formula 2^−(ΔΔCt)^ by the comparative threshold cycle (Ct) method.

### Chromatin Immunoprecipitation Assays

ChIP assays for T24 were performed as previously described (Lee et al., 2006; Sumimoto et al., 2016; Zhang et al., 2016). Briefly, after culturing in normal and glutamine-free culture medium for 3 h, 5×10^6^ cells were crosslinked with 1% formaldehyde for 10 min at room temperature and then washed twice with ice-cold 1 × PBS. The samples were lysed and sonicated to shear crosslinked DNA (25 s ON and 15 s OFF, 39% amplitude, 200 cycles for T24 cells). The Dynal Protein G magnetic beads (Thermo 10003D, Shanghai, China) were preincubated with 1–2 μg of antibodies (c-Jun [60A8] Rabbit mAb, #9165, CST; isotype control mAb, Rabbit [DA1E] mAb IgG, #3900, CST) at 4°C overnight and then incubated with the sheared DNA samples for at least 16 h. The conjugated beads were washed sequentially with 1 ml of low-salt, 1 ml of high-salt, 1 ml of LiCl and 1 ml of TE buffer for 45 min each at 4°C. After reverse-crosslinking and RNase A and proteinase K treatment, DNA was isolated with phenol/chloroform/isoamyl alcohol. RT-qPCR was performed by a Roche LightCycler 480II real-time PCR instrument with SYBR green reagents and the following primers: forward primer, 5′-GTC​ACA​TTT​CAA​GCA​GGA​TGA​CT-3′ and reverse primer, 5′-GAG​TGG​GAA​GGG​GAG​AGA​GT-3′.

### Statistical Analysis

Each experiment was performed at least three times and the data are shown as the mean ± standard deviation (SD). Statistical analyses accompanied by graphs were prepared by GraphPad (La Jolla, CA, United States). Comparisons between two groups were calculated using unpaired t-tests, and multiple comparisons were calculated using 2-way ANOVA. The results were considered significant at *p* < 0.05 (*p < 0.05, **p < 0.01, ***p < 0.001, ****p < 0.0001).

## Data Availability

The original contributions presented in the study are included in the article/Supplementary Material, further inquiries can be directed to the corresponding authors.
