# Supplementary material for: Immunosuppression Induced by Glutamine Deprivation Occurs via Activating PD-L1 Transcription in Bladder Cancer
Source: Front Mol Biosci. 2021 Nov 5;8:687305. doi: 10.3389/fmolb.2021.687305 (PMC8602840; doi:10.3389/fmolb.2021.687305)
Supplement: Supplementary file 1 [file DataSheet1.docx]

Supplementary Material

# Supplementary Figures


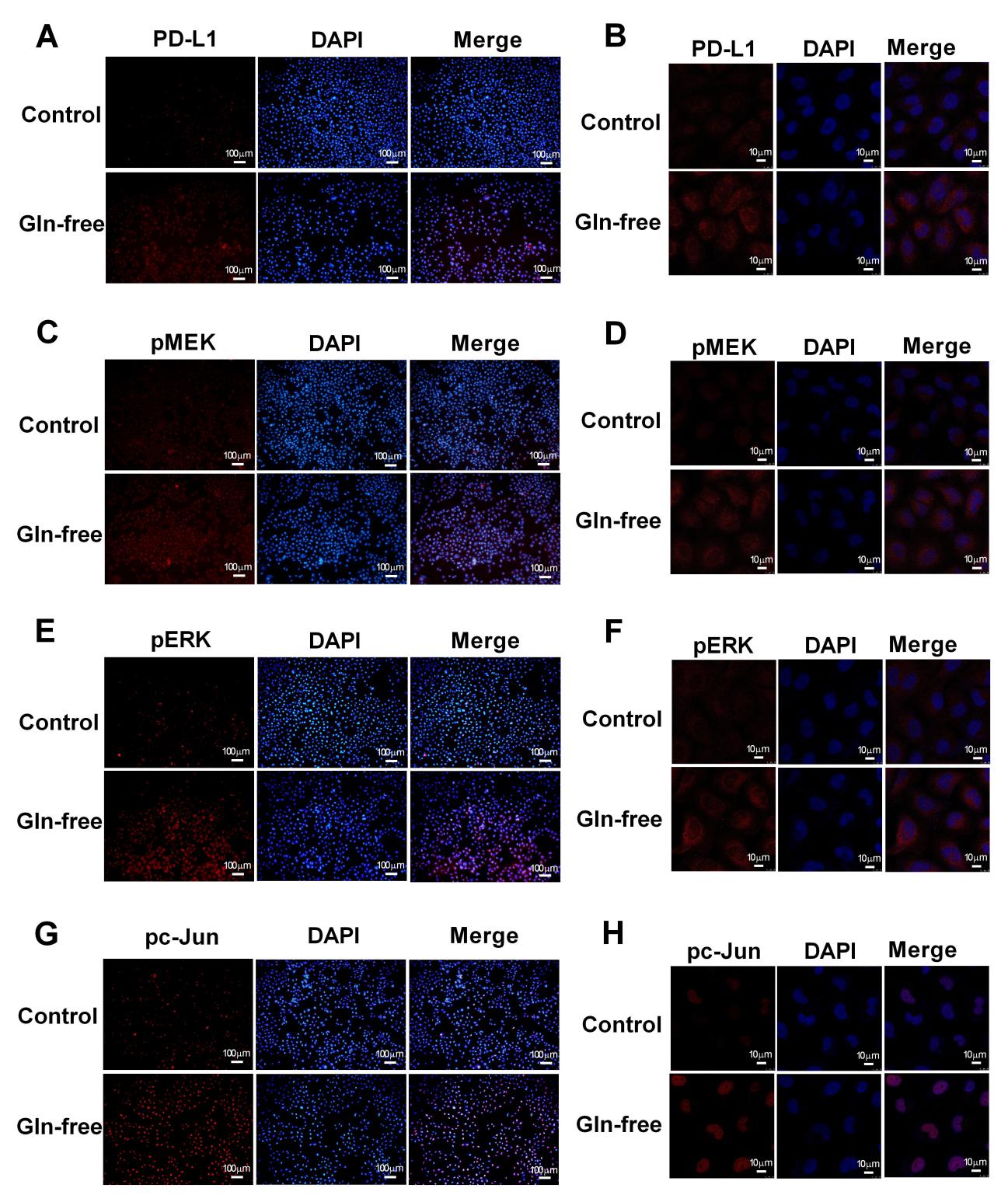


**Supplementary Figure 1.** The PD-L1 expression and MEK/ERK/c-Jun signaling pathway by glutamine deprivation were assessed by immunofluorescence. (**A, C, E, G**) The levels of PD-L1, pMEK, pERK and pc-Jun in glutamine-free medium were assessed by fluorescence microscopy. (**B, D, E, H**) The levels of PD-L1, pMEK, pERK and pc-Jun in glutamine-free medium were assessed by confocal microscopy.


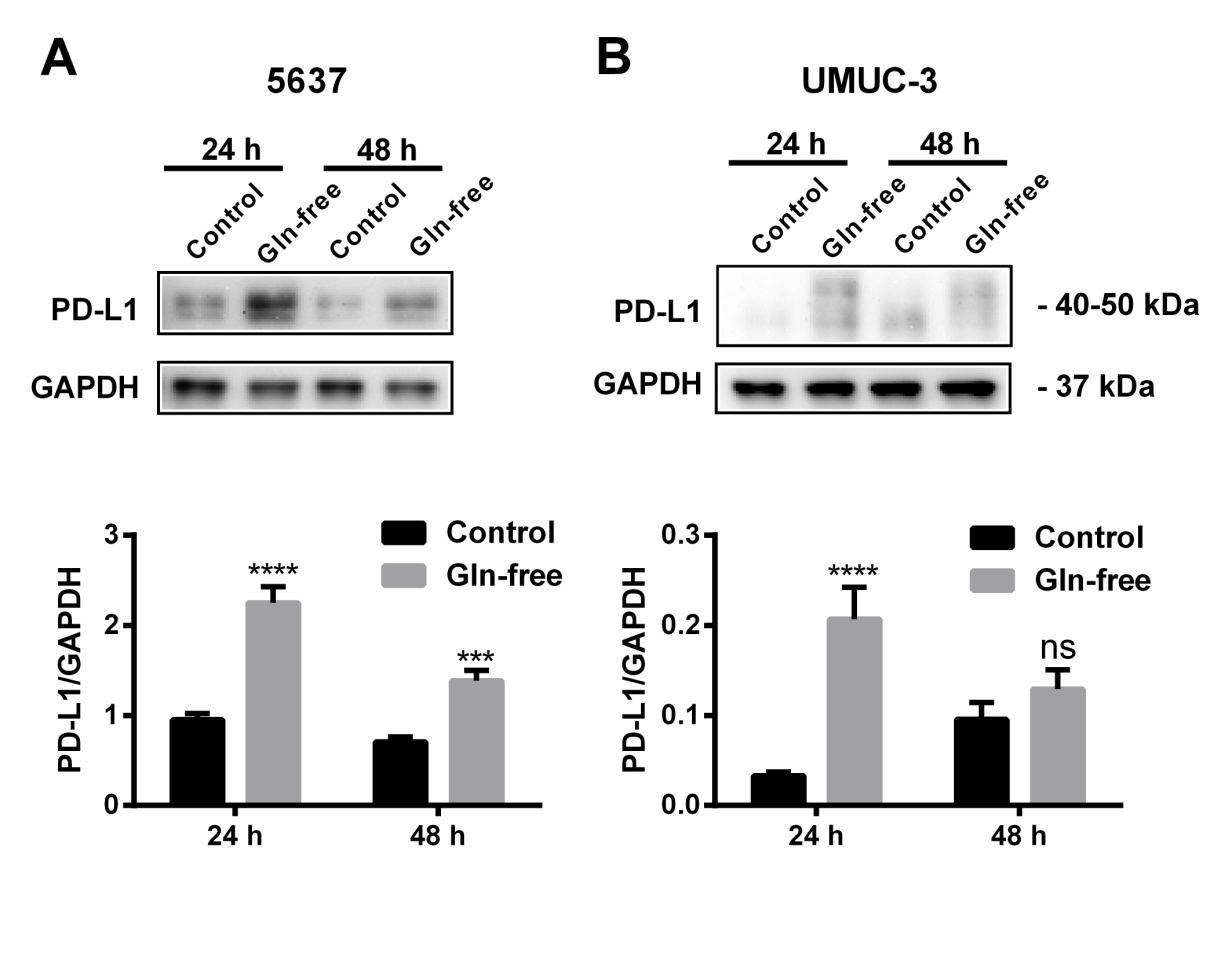


**Supplementary Figure 2.** The PD-L1 expression by glutamine deprivation in 5637 and UMUC-3 cell lines. (**A**) The PD-L1 expression by glutamine deprivation in 5637 at 24 h and 48 h. (**B**) The PD-L1 expression by glutamine deprivation in UMUC-3 at 24 h and 48 h. The densitometric analysis of proteins was performed and the results were normalized to GAPDH. The results were considered significant at p<0.05 (***p<0.001, ****p<0.0001).


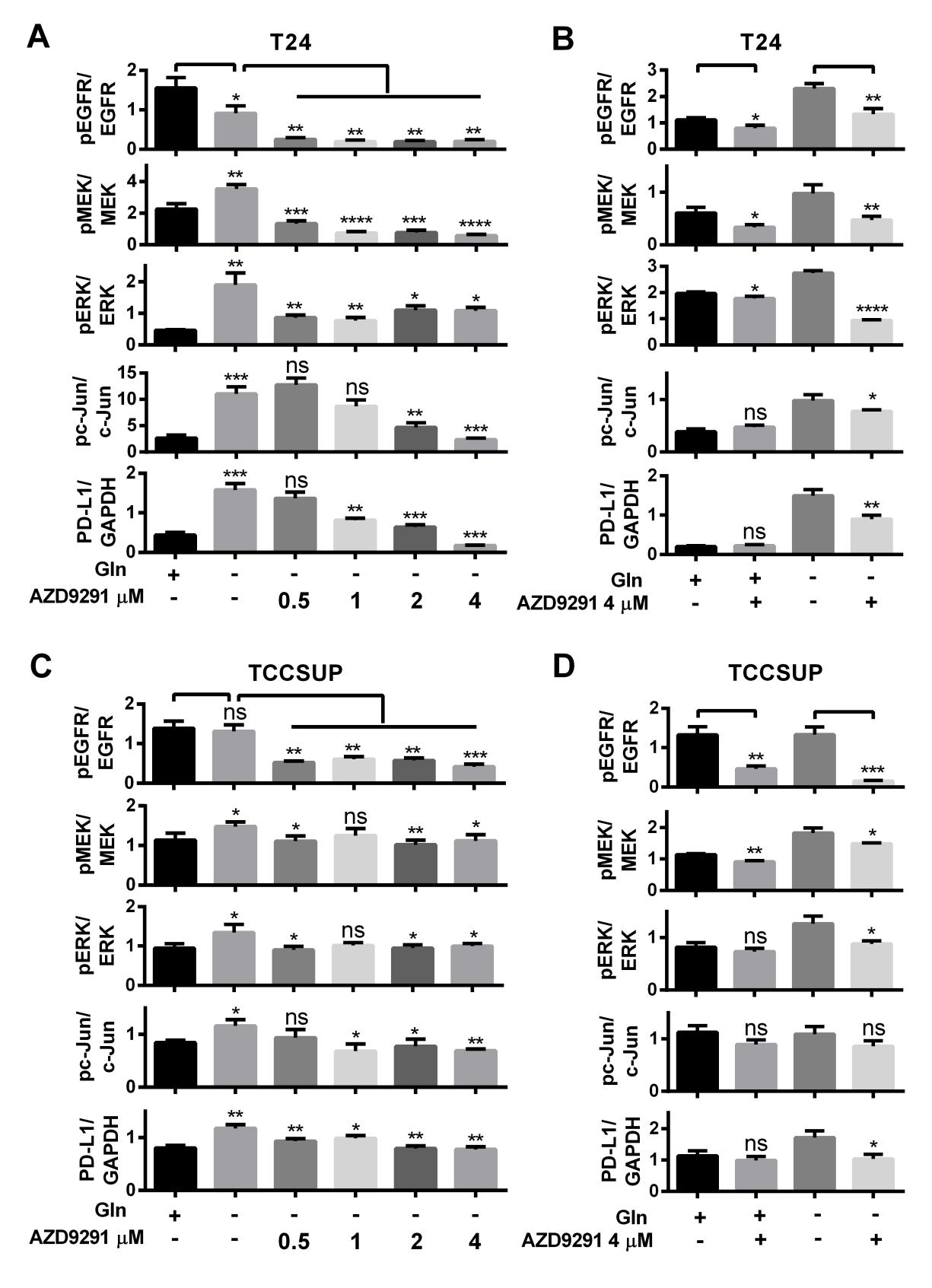


**Supplementary Figure 3.** The protein quantification analysis of figure 3. Inhibiting EGFR activation can reduce the elevated PD-L1 level induced by glutamine deprivation in T24 and TCCSUP cells. (**A**) The protein quantification analysis of pEGFR/EGFR, pMEK/MEK, pERK/ERK, pc-Jun/c-Jun and PD-L1/GAPDH in figure 3A. (**B**) The protein quantification analysis of pEGFR/EGFR, pMEK/MEK, pERK/ERK, pc-Jun/c-Jun and PD-L1/GAPDH in figure 3B. (**C**) The protein quantification analysis of pEGFR/EGFR, pMEK/MEK, pERK/ERK, pc-Jun/c-Jun and PD-L1/GAPDH in figure 3C. (**D**) The protein quantification analysis of pEGFR/EGFR, pMEK/MEK, pERK/ERK, pc-Jun/c-Jun and PD-L1/GAPDH in figure 3D. Statistical analysis was performed between control group and Gln-free group, as well as Gln-free group and inhibitor group in A&C. In B&D, statistical analysis was performed between control group and inhibitor group in normal or Gln-free medium, respectively. The results were considered significant at p<0.05 (*p<0.05, **p<0.01, ***p<0.001, ****p<0.0001).


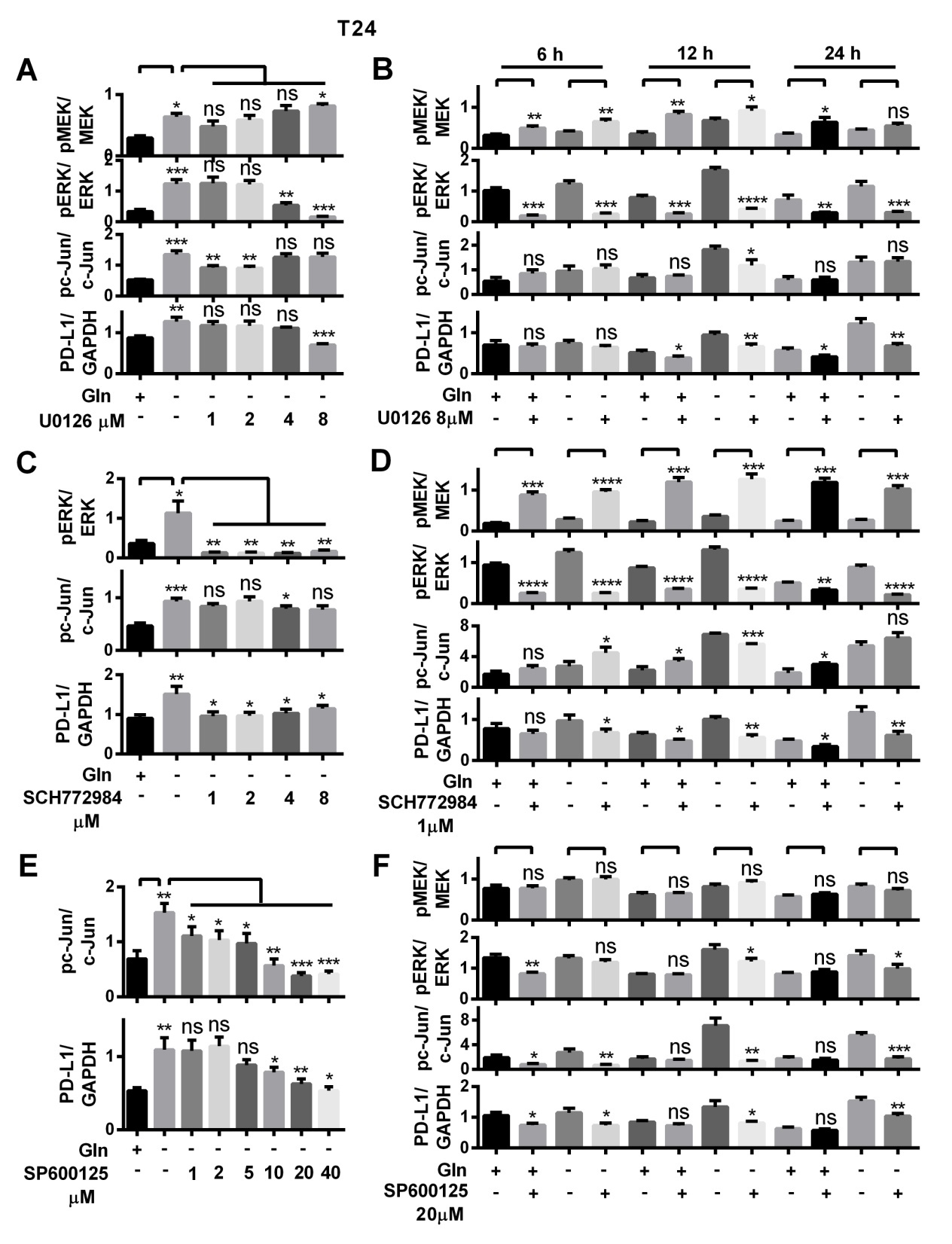


**Supplementary Figure 4.** The protein quantification analysis of figure 4. Inhibiting pMEK, pERK or pc-Jun activation can reduce the elevated PD-L1 level induced by glutamine deprivation in T24 cells. (**A**) The protein quantification analysis of pMEK/MEK, pERK/ERK, pc-Jun/c-Jun and PD-L1/GAPDH in figure 4A. (**B**) The protein quantification analysis of pMEK/MEK, pERK/ERK, pc-Jun/c-Jun and PD-L1/GAPDH in figure 4B. (**C**) The protein quantification analysis of pERK/ERK, pc-Jun/c-Jun and PD-L1/GAPDH in figure 4C. (**D**) The protein quantification analysis of pMEK/MEK, pERK/ERK, pc-Jun/c-Jun and PD-L1/GAPDH in figure 4D. (**E**) The protein quantification analysis of pc-Jun/c-Jun and PD-L1/GAPDH in figure 4E. (**F**) The protein quantification analysis of pMEK/MEK, pERK/ERK, pc-Jun/c-Jun and PD-L1/GAPDH in figure 4F. Statistical analysis was performed between control group and Gln-free group, as well as Gln-free group and inhibitor group in A&C&E. In B&D&F, statistical analysis was performed between control group and inhibitor group in normal or Gln-free medium respectively, at different time points. The results were considered significant at p<0.05 (*p<0.05, **p<0.01, ***p<0.001, ****p<0.0001).


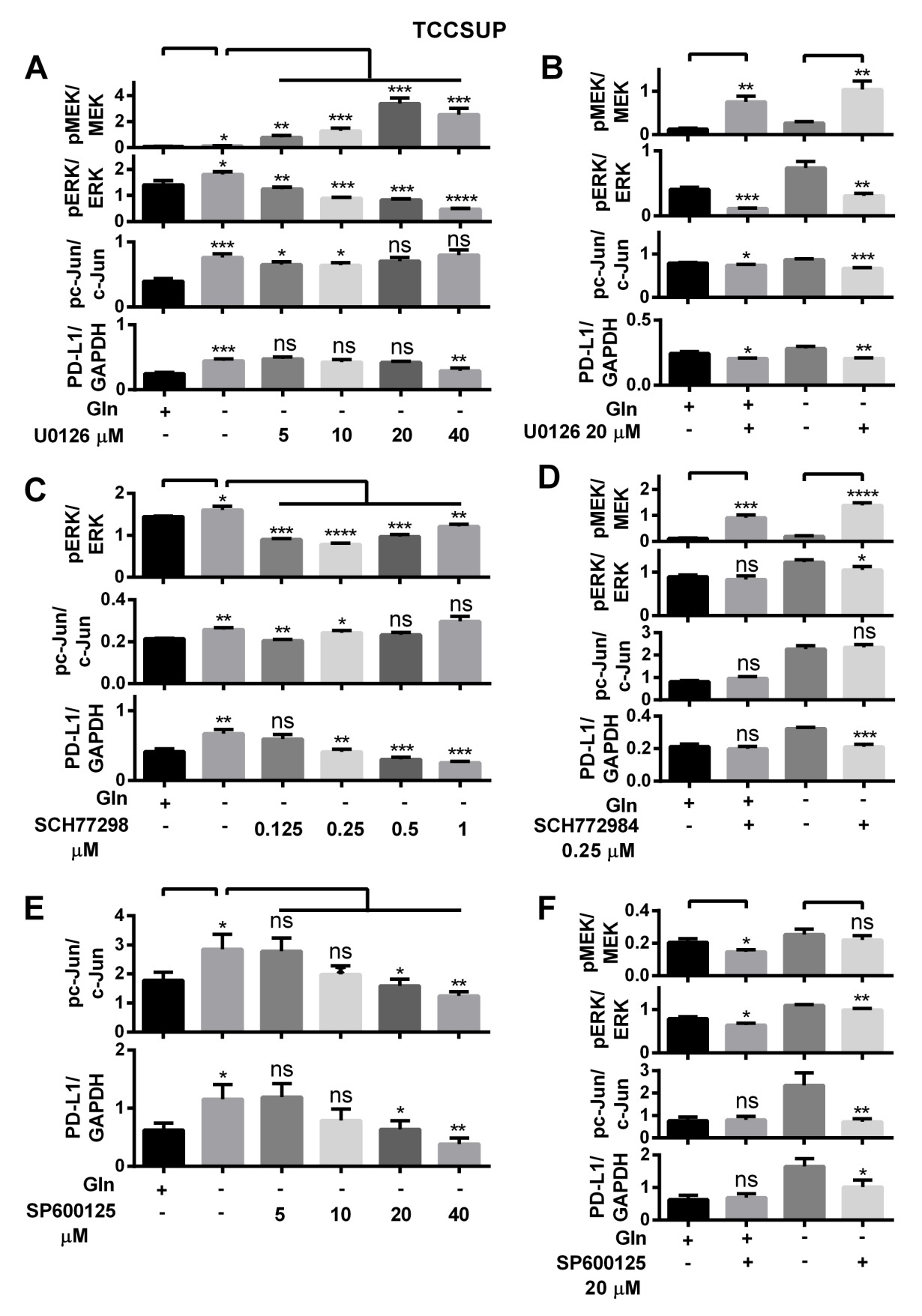


**Supplementary Figure 5.** The protein quantification analysis of figure 5. Inhibiting pMEK, pERK or pc-Jun activation can reduce the elevated PD-L1 level induced by glutamine deprivation in TCCSUP cells. (**A**) The protein quantification analysis of pMEK/MEK, pERK/ERK, pc-Jun/c-Jun and PD-L1/GAPDH in figure 5A. (**B**) The protein quantification analysis of pMEK/MEK, pERK/ERK, pc-Jun/c-Jun and PD-L1/GAPDH in figure 5B. (**C**) The protein quantification analysis of pERK/ERK, pc-Jun/c-Jun and PD-L1/GAPDH in figure 5C. (**D**) The protein quantification analysis of pMEK/MEK, pERK/ERK, pc-Jun/c-Jun and PD-L1/GAPDH in figure 5D. (**E**) The protein quantification analysis of pc-Jun/c-Jun and PD-L1/GAPDH in figure 5E. (**F**) The protein quantification analysis of pMEK/MEK, pERK/ERK, pc-Jun/c-Jun and PD-L1/GAPDH in figure 5F. Statistical analysis was performed between control group and Gln-free group, as well as Gln-free group and inhibitor group in A&C&E. In B&D&F, statistical analysis was performed between control group and inhibitor group in normal or Gln-free medium respectively. The results were considered significant at p<0.05 (*p<0.05, **p<0.01, ***p<0.001, ****p<0.0001).


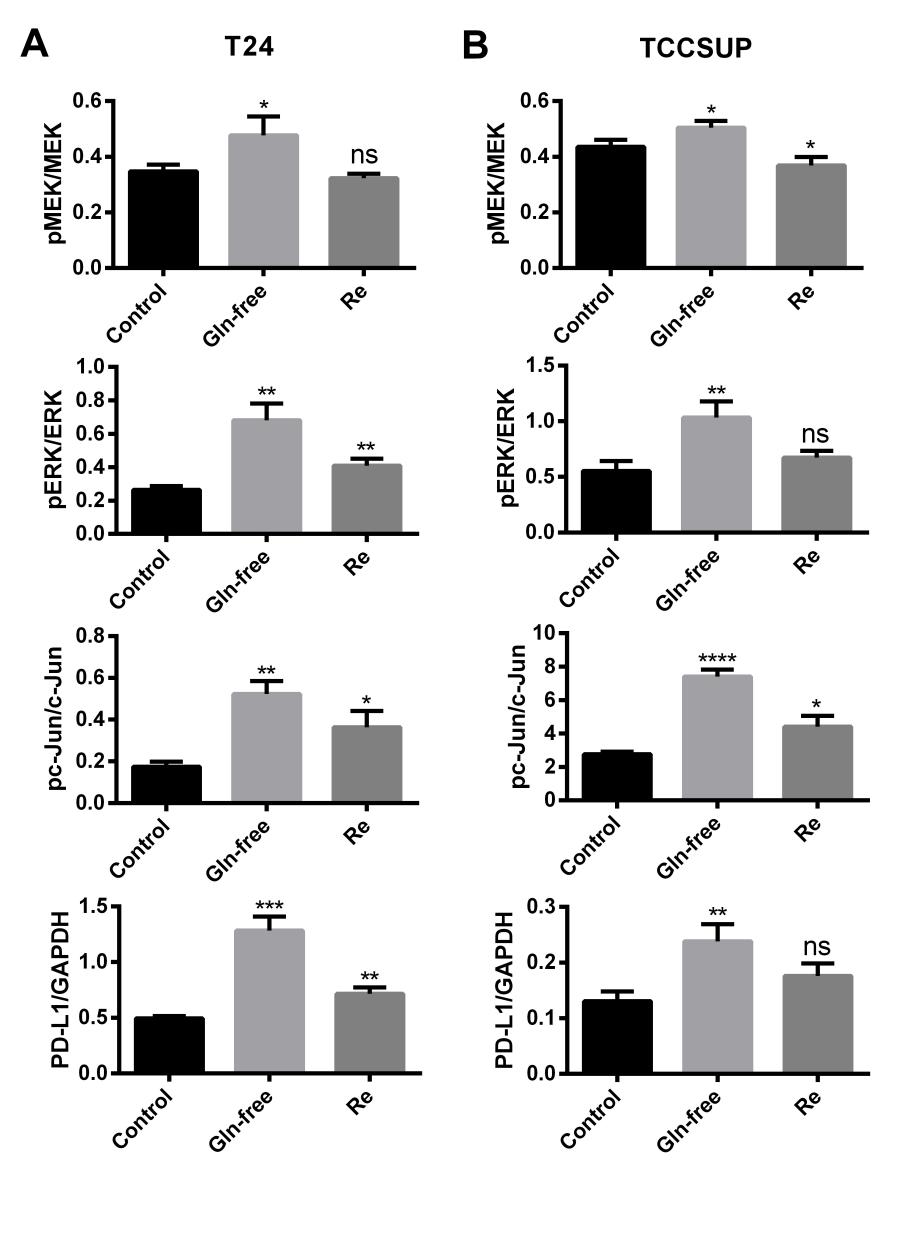


**Supplementary Figure 6.** The protein quantification analyses of figure 7A&B. Upregulation of PD-L1 and the MEK/ERK/c-Jun pathway were reduced after glutamine recovery. (**A**) The protein quantification analysis of pMEK/MEK, pERK/ERK, pc-Jun/c-Jun and PD-L1/GAPDH in figure 7A. (**B**) The protein quantification analysis of pMEK/MEK, pERK/ERK, pc-Jun/c-Jun and PD-L1/GAPDH in figure 7B. (Re: glutamine recovery). Statistical analysis was performed between control group and Gln-free group, as well as control group and glutamine recovery group. The results were considered significant at p<0.05 (*p<0.05, **p<0.01, ***p<0.001, ****p<0.0001).


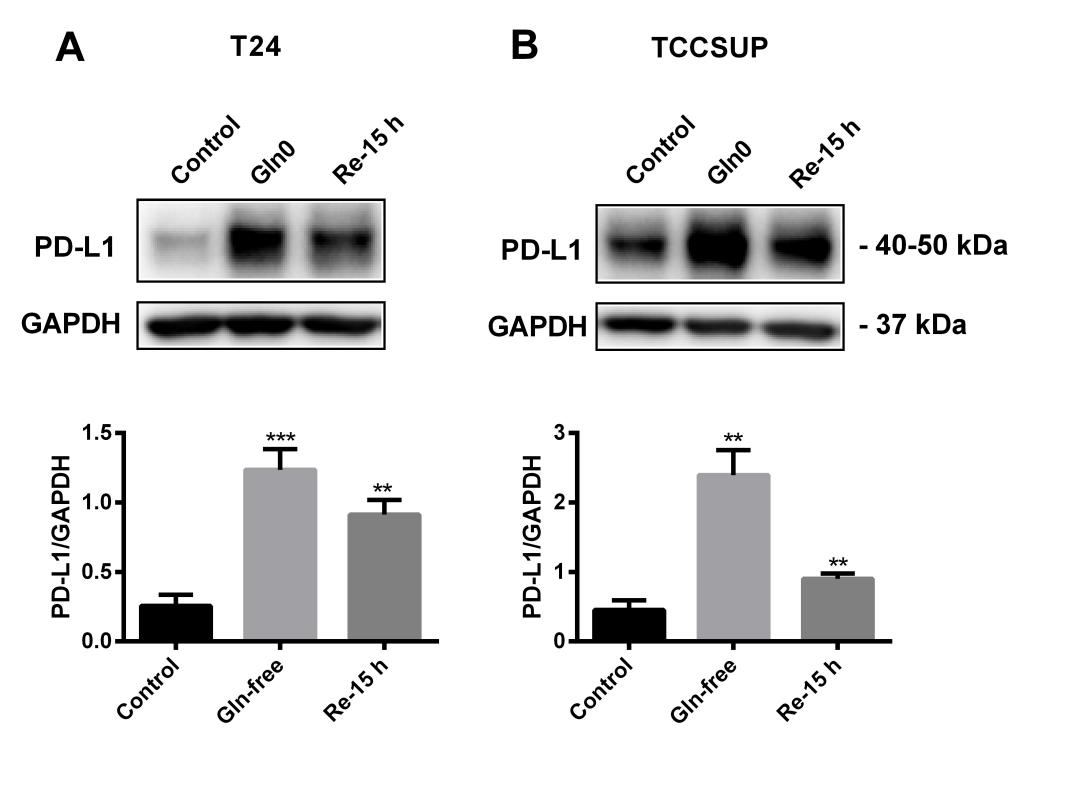


**Supplementary Figure 7.** PD-L1 expression after glutamine recovery for 15 h. (**A**) PD-L1 expression after glutamine recovery for 15 h in T24. (**B**) PD-L1 expression after glutamine recovery for 15 h in TCCSUP. (Re-15 h: glutamine recovery for 15 h). The densitometric analysis of proteins was performed and the results were normalized to GAPDH. The results were considered significant at p<0.05 (**p<0.01, ***p<0.001).


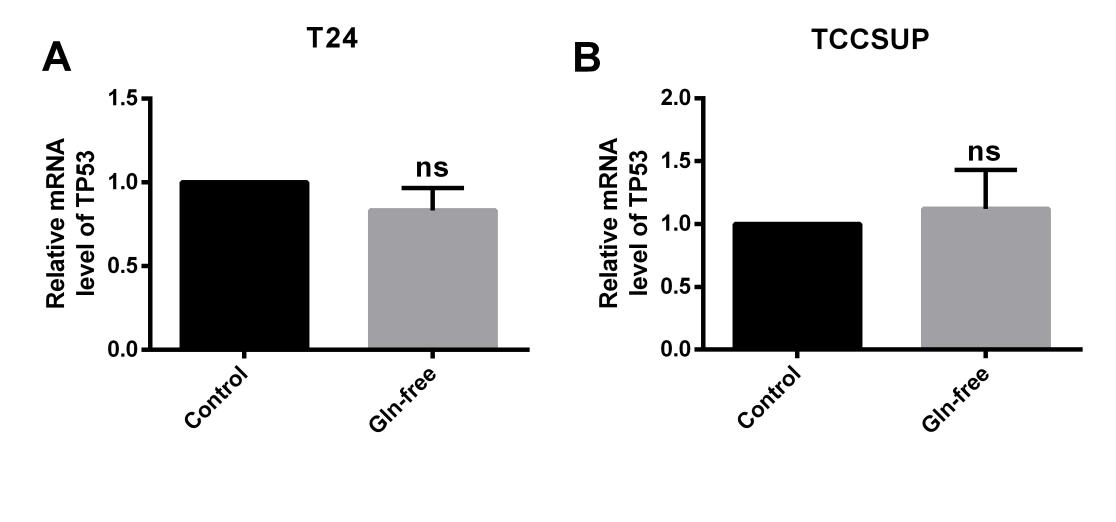


**Supplementary Figure 8.** The mRNA levels of p53 by glutamine deprivation for 24 h. (**A**) The mRNA levels of p53 by glutamine deprivation for 24 h in T24. (**B**) The mRNA levels of p53 by glutamine deprivation for 24 h in TCCSUP.
